# Coronavirus Disease 2019 (COVID-19) and Ecchymosis: A Case Report

**DOI:** 10.7759/cureus.23326

**Published:** 2022-03-19

**Authors:** Jing Hong Fong, Sabrina Mei Ann Koay, Edwin Hsiang Win Foo, Yew Leng Mah, Wei Cheong Eu

**Affiliations:** 1 Department of Emergency Medicine, North West Regional Hospital, Burnie, AUS; 2 Department of Orthopaedics and Traumatology, Penang General Hospital, George Town, MYS; 3 Department of Internal Medicine/Department of Orthopaedics and Traumatology, Hospital Sultanah Bahiyah, Alor Setar, MYS

**Keywords:** covid and skin, pericarditis, non allergic rash, rash, vasculopathy, cutaneous manifestation, covid 19, ecchymosis

## Abstract

Extrapulmonary symptoms such as cutaneous manifestations are increasingly being reported in coronavirus disease 2019 (COVID-19) infection. The rashes of COVID-19 are usually livedo and purpuric and have been classified into six main patterns. This is the first case reported in Malaysia of ecchymosis associated with COVID-19 occurring on a patient without prior history of coagulopathy. The report of this rare clinical association could have a potential pathophysiological implication and contributes to the current data of the cutaneous manifestations of COVID-19. Further knowledge regarding these cutaneous lesions could help in the diagnosis of COVID-19 related complications and earlier management.

## Introduction

In March 2020, World Health Organisation (WHO) announced coronavirus disease 2019 (COVID-19), a newly emerged disease caused by novel RNA virus - severe acute respiratory syndrome coronavirus 2 (SARS-CoV-2), a pandemic emergency [1,2]. Extrapulmonary symptoms of COVID-19, particularly of cutaneous manifestations, have been increasingly reported but pulmonary symptoms like cough, hyposmia, and dyspnoea remain common [1,2]. These dermatological patterns include: (i) urticarial rash, (ii) confluent erythematous/maculopapular/morbilliform rash, (iii) papulovesicular exanthem, (iv) chilblain-like acral pattern, (v) livedo reticularis, (vi) purpuric vasculitic pattern [2].

To date, studies have shown that these skin manifestations differ in terms of patients' demography (including age, geography), pathophysiological mechanism, as well as disease timeline and severity [3]. Indeed, livedo and purpuric rashes commonly affect the elderly population and carry a higher prevalence of hospitalization and mechanical ventilation [3], while the chilblain-like acral pattern mostly affects Caucasian groups and those without systemic symptoms [3]. Hence, further knowledge regarding these cutaneous lesions could help in the diagnosis of COVID-19. 

## Case presentation

We are reporting a case of a 58-year-old Malaysian Chinese woman, who is a nursing home resident with a medical history of type 2 diabetes mellitus (T2DM), hypertension, and a history of hemorrhagic stroke. She also had a left parietal craniectomy done in 2016 and is complicated with left-sided residual weakness and limbs contracture. Initially presented to the emergency department of Penang General Hospital, Malaysia, with the chief complaints of lethargy, poor oral intake, and diarrhea for one week. Due to a high level of suspicion, the SARS-CoV-2 real-time polymerase chain reaction (RT-PCR) swab was performed, which turned out to be positive. She was subsequently admitted to the COVID-19 isolation ward for further monitoring and treatments.

On day four of admission, her clinical condition deteriorated, she required a high flow nasal canula of 60L/60%, and her chest x-ray showed peripheral infiltration with ground-glass opacity. She was treated under the impression of cytokine release syndrome (CRS) caused by COVID-19 and was started on steroid therapy, thromboprophylaxis, and empirical antibiotics. Her condition was further complicated with acute pericarditis, for which she required oral colchicine in addition to her medical therapy.

**Figure 1:**
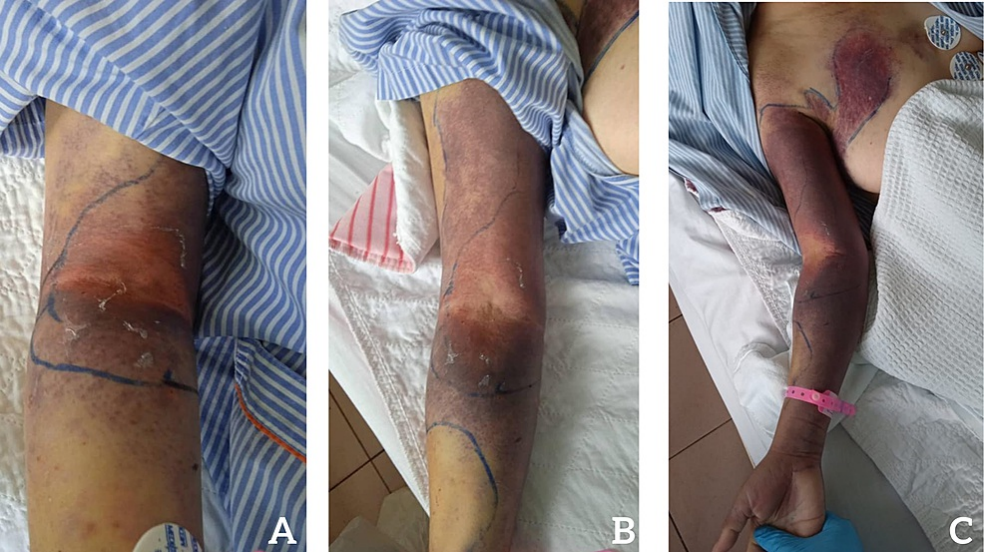
The extension of swelling and ecchymosis from the right cubital fossa on (A) day seven, (B) day ten, and (C) day eleven. The swollen area is outlined using a marker.

**Figure 2:**
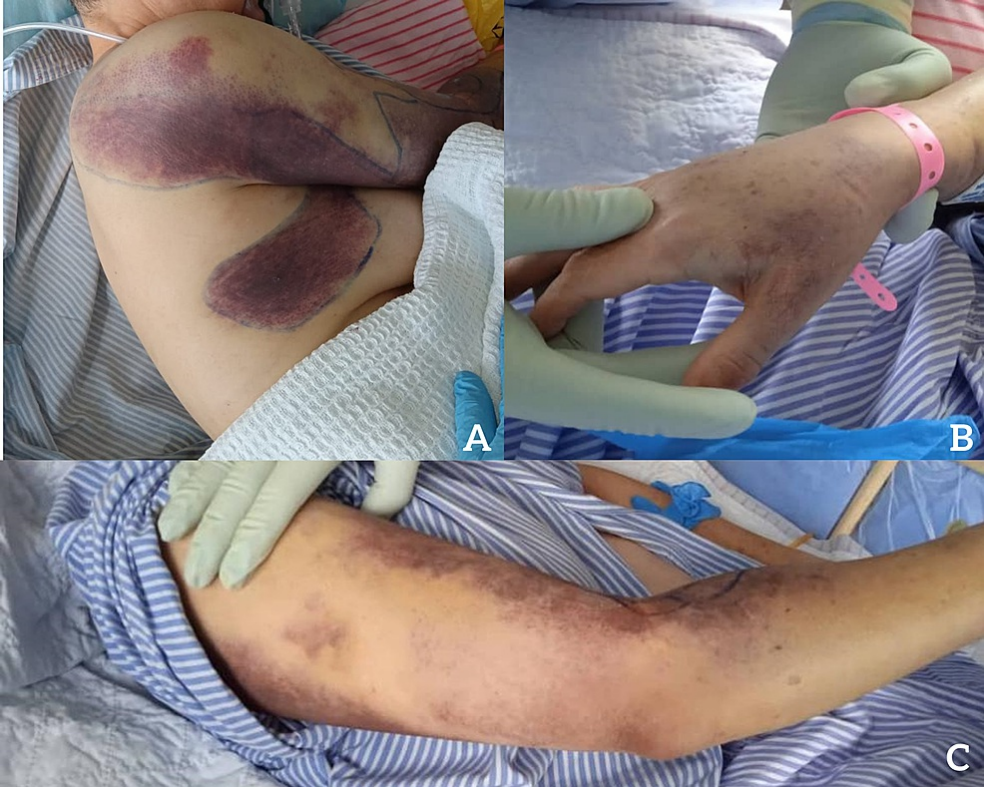
(A) Right lateral view showing the extension of the ecchymosis to the upper back of the patient; (B) purpuric rash on the dorsal of the right hand; (C) purpuric rash on the medio-posterior of forearm.

On day seven of admission, a painful, erythematous, swollen area was noted over her right cubital fossa, with the rapid expansion of ecchymosis to the antero-medio-posterior aspect of the forearm, right anterior chest, and upper back on day 10 of admission (Figure 1). She was also noted to have purpura on the dorsal area of her right hand (Figure 2). On examination, tenderness was reported from the cubital fossa up to mid-arm level. Examinations to rule out compartment syndrome was done. Her capillary refill time (CRT) of all fingers on the right hand was three seconds, radial pulse was not palpable, brachial pulse was feeble, anterior and posterior compartments were soft. Passive stretch test (pain with passive stretching of muscles) was negative. It was unlikely that she had compartment syndrome. The range of motion over the right elbow joint was mildly restricted due to pain. Thromboprophylaxis was immediately withheld. Her hemoglobin (Hb) dropped from 14 g/dL to 8.2 g/dL, requiring a blood transfusion. Selected results of related blood investigations are given in Table 1. Computed tomography angiography (CTA) of the right upper limb was done urgently to identify the presence and cause of active bleeding, particularly from the brachial artery.

**Table 1:** Selected results of related blood investigations.

Investigation	Unit	Normal Range	Day of Admission					
			4	6	7	10	12	16
Total white cell count	10^3^/μL	4.0 - 10.0	2.8	10	15	19	16	13
Hemoglobin	g/dL	12.0 - 15.0	14	11	8.2	9.9	11	12
Platelet	10^3^/μL	150 - 410	122	185	220	411	283	200
C-reactive protein	mg/L	< 5.0	39		14	25.1	17	35
Prothrombin time	seconds	9.4 - 11.0			9.9			10
Activated partial thromboplastin clotting time	seconds	22.2 - 31.0			26			22
International normalized ratio	ratio	0.90 - 1.10			1			1
Creatinine kinase	U/L	< 170		159	161			
Blood culture						No growth		

**Figure 3:**
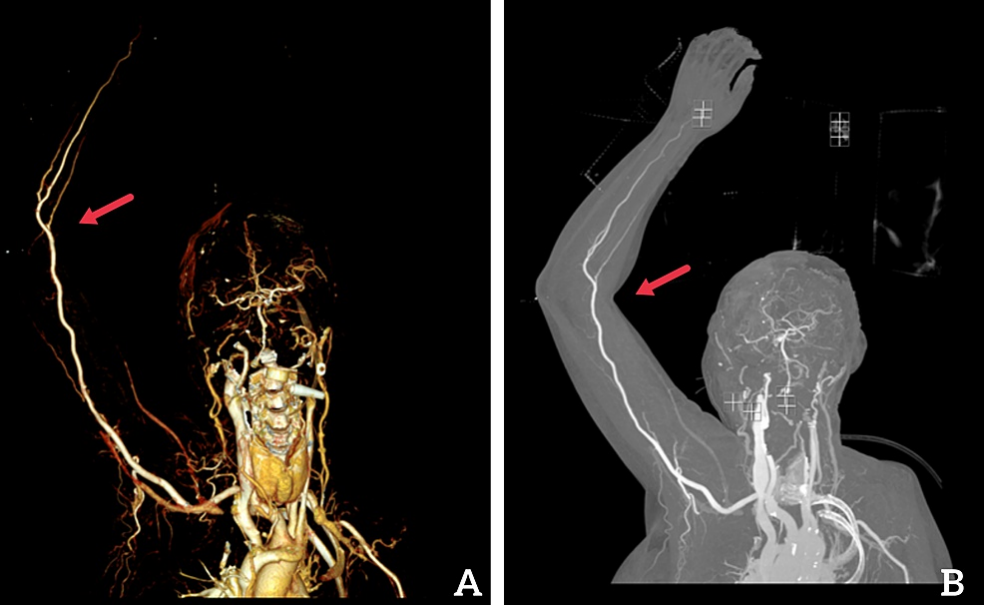
Computed tomography angiogram (CTA) of right upper extremity: (A) three-dimensional reconstruction view, (B) coronal reconstruction view. Red arrow indicates the location of the hematoma.

CTA showed rim enhancing collection over the inter-fascial plane in between the biceps brachii muscle and subcutaneous tissue, extending from the elbow to the arm, measuring 5.2cm x 0.7cm x 8.4cm, suggestive of infected hematoma (Figure 3). The imaging also showed myositis of adjacent biceps brachii muscle, and extensive cellulitis from the forearm up to the right axillary region. Intravenous (IV) piperacillin/tazobactam 4.5g four times a day was then started empirically and continued for one week. The right upper limb was elevated, with ice compression, and closed monitoring of its neurovascular status was done. No surgical evacuation of hematoma was done, no evidence of compartment syndrome throughout the stay, the size of hematoma and ecchymosis slowly and gradually decreased, pain over the right upper limb much improved.

Her general condition gradually improved. She was able to wean off oxygen therapy on day 15 of admission and was successfully discharged from the ward on day 18 of admission. Upon discharge, she was comfortable in room air, with no shortness of breath or chest pain. Upon examination of the right upper limb, the area of redness was much smaller, extending from cubital fossa to distal forearm level, not warm to touch, and no longer tender. There was no restriction of the range of motion over the right upper limb. Distal pulses were palpable, and all fingers were pink with CRT <2 seconds.

## Discussion

This is the first case report on a large, widespread ecchymosis occurring on a patient after the diagnosis of COVID-19 infection and without the association of coagulopathies.

Of COVID-19 cases, 0.6-20.4% reported dermatological lesions, suggesting them as a sign of the disease [4]. Our report is consistent with previous studies regarding the timeline of purpura rash appearance, which is during week 0-4 (acute and post-acute) phase of COVID-19 [5]. Such rash reflects a complement-mediated endothelial injury, involving the deposition of C5b-9 and C4d components within cutaneous microvasculature [6]. Nevertheless, the various COVID-19 medications such as camostat mesylate used are also associated with cutaneous side effects [1]. Careful interpretation of rash is needed to differentiate it from treatment drug adverse effects, other local viral illnesses (eg dengue, immune response to viral nucleotide), and secondary dermatological reaction due to systemic effect of COVID-19 (eg. immune thrombocytopenic purpura) [7]. The ecchymosis of the case is unlikely caused by immune thrombocytopenic purpura or other coagulopathies as the patient’s platelet counts were within normal range. 

Besides that, a recent study from the United Kingdom (UK) showed that rashes, as compared to fever, have a more specific diagnostic value of COVID-19 (odds ratio 1.67) [1]. In view of a high level of false-negative results from the COVID-19 PCR/serology test, rashes could have become a possible future diagnostic criterion. This is particularly important and could help in areas with limited access to laboratory diagnostic tools [8].

The appearance of ecchymosis three days after our patient was placed on higher oxygen delivery, correlates with reports of such vascular rash in critically ill patients, often carrying a bad prognosis [3] As purpuric rashes have mostly resulted from cutaneous thrombosis, they could indicate vascular occlusion and thrombosis in other organs [3]. Poor clinical awareness has, however, resulted in the late detection of these crucial rashes. Hence, early identification is essential to allow prompt aggressive treatment and to prevent end-organ failure risk and death. 

Of note, our study only explores the cutaneous manifestation of COVID-19 in an individual case. Larger-scale studies are preferably needed to understand more on the various rashes and their significance in terms of both diagnostic and prognostic value for COVID-19 patients. Here, our patient fortunately survived and was discharged from the COVID-19 pathway. Following up on these patients allows us to further explore and understand the implication of rashes in the post-COVID-19 recovery pathway.

## Conclusions

Data on cutaneous associations related to COVID-19 is still limited and non-specific. This is the first case report of ecchymosis associated with COVID-19 infection occurring on a patient without a prior history of coagulopathy. The report of this rare clinical association could be a potential pathophysiological implication and contributes to the current data of the cutaneous manifestations of COVID-19.
